# Forecasting tuberculosis epidemics using an autoregressive fractionally integrated moving average model: a 17-year time series analysis

**DOI:** 10.7189/jogh.15.04208

**Published:** 2025-07-25

**Authors:** Yongbin Wang, Yifang Liang, Bingjie Zhang, Shibei Yi, Peiping Zhou, Xianxiang Lan, Chenlu Xue, Yanyan Li, Xinxiao Li, Chunjie Xu

**Affiliations:** 1 Department of Epidemiology and Health Statistics, School of Public Health, Xinxiang Medical University, Xinxiang, China; 2 Beijing Key Laboratory of Antimicrobial Agents/Laboratory of Pharmacology, Institute of Medicinal Biotechnology, Chinese Academy of Medical Sciences & Peking Union Medical College, Beijing, China

## Abstract

**Background:**

Tuberculosis (TB) remains a significant public health challenge in Henan, China, requiring accurate forecasting to guide prevention and control efforts. While traditional models like autoregressive integrated moving average (ARIMA) are commonly used, they may not fully capture long-term dependencies in the data. This study evaluates the autoregressive fractionally integrated moving average (ARFIMA) model, which incorporates fractional differencing, to improve TB forecasting by better modelling long-range dependencies and seasonal patterns.

**Methods:**

Monthly TB incidence data from January 2007 to May 2023 in Henan were collected. The data set was split into a training set (January 2007–May 2022) and a test set (June 2022–May 2023). Both ARIMA and ARFIMA models were developed using the training set, and their predictive accuracy was assessed on the test set using metrics such as mean absolute deviation, mean absolute percentage error, mean square error, and mean error rate. A sensitivity analysis was conducted to evaluate the robustness of the forecasts.

**Results:**

There were 1 074 081 TB incident cases in Henan during the study period. The TB incidence was reducing at an annual rate of 5.83%, with the seasonal factor >1 between March–July and seasonal factor <1 in other months. The ARIMA (2,0,1)(0,1,1)_12_ and ARFIMA (2,0,1)(0,0.38,1)_12_ models were identified as suitable for the data. The ARFIMA model consistently outperformed ARIMA model in the forecasting phase, with lower errors across all metrics (*e.g*. mean absolute deviation: 467 *vs*. 569.54; mean absolute percentage error: 0.19 *vs*. 0.21; mean square error: 620.48 *vs*. 690.11; mean error rate: 0.14 *vs*. 0.17). This indicated that the ARFIMA model better captures long-term dependencies and seasonal patterns, leading to more accurate forecasts.

**Conclusions:**

Tuberculosis incidence in Henan shows a clear downward trend with distinct seasonal variation. The ARFIMA model provides more accurate TB incidence forecasts than ARIMA, particularly in capturing long-term trends and seasonality. Effective management of TB at the population level requires proper monitoring and understanding of disease patterns. Forecasting serves as a critical tool for detecting deviations from expected trends, which may signal changes in disease dynamics. Continuous use of the ARFIMA model is essential for guiding public health interventions and ensuring timely responses to emerging challenges in TB control.

Tuberculosis (TB) is a bacterial infection caused by the pathogen *Mycobacterium tuberculosis* [1]. Despite significant global health advancements, TB remains the 13th leading cause of death and the second leading infectious killer after COVID-19, surpassing even HIV/AIDS. The consequences of TB are devastating for individuals, communities, and economies. Between 2015–2021, global efforts resulted in approximately 2 and 5.9% reductions in TB morbidity and mortality, respectively [1]. In 2023, an estimated 10.8 million individuals developed TB globally, with around 1.6 million deaths attributed to the disease [1]. Alarmingly, 87% of new cases were reported in 30 high-burden countries, with China accounting for the third highest TB burden (6.8%) and ranking second for drug-resistant TB. Although China has witnessed a 3% annual decline in TB morbidity since 2005 [1], progress has stagnated or even reversed due to various public health threats, including the rise of drug-resistant TB, co-infection with HIV, overcrowding, malnutrition, emerging infectious diseases, population mobility, and the coexistence of TB with chronic noncommunicable diseases such as diabetes and hypertension [1–3]. These challenges jeopardise the milestones and targets set for ending the TB epidemic. Given that TB is both preventable and treatable, there is an urgent need for multifaceted responses to combat this life-threatening disease. A cornerstone of any effective TB control strategy is the ability to accurately forecast epidemic trends [4], which can inform the intensity and type of interventions required to contain the spread of the disease [4].

Time series analysis offers valuable insights into the dynamics of various diseases, enabling predictions of observed phenomena and the establishment of quality control systems [5]. The temporal variations of infectious diseases are influenced by multiple factors, leading to epidemic characteristics such as seasonality, secular trend, periodicity, and randomness [6,7]. Understanding these dynamics is crucial for developing effective disease control and prevention strategies. The selection of an appropriate forecasting model is pivotal, not only for theoretical accuracy but also for practical applicability in public health strategy formulation. Among the quantitative methods available for time series forecasting, the autoregressive integrated moving average (ARIMA) and autoregressive fractionally integrated moving average (ARFIMA) models stand out as two prominent paradigms. The ARIMA model, known for its simplicity, fast applicability, and effectiveness, has been the most popular tool in estimating infectious diseases epidemics, including TB, by accounting for changing trends, periodic variations, and random fluctuations within the data [5,8–13]. However, infectious disease epidemics frequently exhibit persistent dependencies over longer time lags [7]. While the ARIMA model has been successfully applied, it falls short in adequately capturing long-run temporal dependencies [14]. This limitation arises from its reliance on integer differencing to achieve stationarity, which can lead to over-differencing and the loss of valuable information that may affect parameter estimation and model fitting [7]. In contrast, the ARFIMA model extends the ARIMA framework by allowing for fractional differencing in time series data [14,15]. This feature enables the ARFIMA model to effectively model long-range dependence, a phenomenon frequently observed in the incidence of infectious diseases due to socio-environmental factors and historical contagion patterns. By capturing the inherent dependency features in the data and distinguishing between short-run fluctuations and long-term trends, the ARFIMA model offers a more flexible modelling approach that can yield improved predictions for time series characterised by persistent dependencies [14,16–18]. However, it remains unclear whether the ARFIMA model is well-suited to analyse and forecast TB epidemics. To do this, this study aims to investigate the flexibility and efficacy of the ARFIMA model in estimating the temporal trends of TB and to compare its predictive performance with that of the ARIMA model.

## METHODS

### Data sources

This population-based observational study retrospectively collected monthly TB incident cases from January 2007 to May 2023. The data were sourced from the Health Commission of Henan [19] and the National Population and Health Science Data Sharing Platform [20]. Population data were obtained from Henan Statistical Yearbook. All TB cases were confirmed by qualified health care professionals according to the diagnostic criteria established by the National Health Commission of the People's Republic of China [21]. As TB is classified as a notifiable disease in China, all confirmed cases must be reported within 24 hours, with repeated records corrected by the end of the same month.

### Building the ARIMA model

The ARIMA model comprises three main terms: autoregression (AR), integration (I), and moving average (MA) [22]. For seasonal data, three additional parameters are introduced: seasonal AR, seasonal integration, and seasonal MA [22]. By combining both non-seasonal and seasonal parts, the ARIMA model can capture underlying patterns and dynamics in TB incidence, including trends, seasonality, and irregularities. The ARIMA model is typically represented as (p, d, q) × (P, D, Q)_S_, where (p, d, q) denotes the non-seasonal orders of the AR, I, and MA; (P, D, Q) signifies the seasonal orders of the counterparts; and s represents the length of the seasonal cycle (*e.g*. 12 for monthly series, seven for weekly series) [4]. The processes of establishing an ARIMA model involves four steps [4,9–13]:

1. Stationarity check. The ARIMA model assumes that the series is stationary. Thus, we assessed the stationarity of the TB incidence series using the Augmented Dickey-Fuller (ADF) test. If the ADF statistic indicated non-stationarity, differencing was applied to achieve stationarity.

2. Parameters identification and estimation. The potential orders of the key parameters p, q, P, and Q were initially selected through autocorrelation function (ACF) and partial autocorrelation function (PACF) analyses. The optimal model was then determined by minimising the Bayesian information criterion (BIC), Akaike information criterion (AIC), or corrected AIC.

3. Model diagnosis. The significance of parameters and the randomness of residuals (*i.e*. white noise series) were assessed using t-statistics, ACF, PACF, and Ljung-Box Q statistics. A model was deemed adequate if no significant autocorrelations existed beyond the 95% confidence interval (CI) and if the Ljung-Box test yielded a *P*-value >0.05.

4. Forecasting. Once the preferred model passed all the statistical checks, it could be fitted to the TB series to generate predictions.

### Building the ARFIMA model

Time series data often exhibit persistent dependencies over longer lags, a phenomenon known as hyperbolic decay [7,23]. The ARFIMA model is currently the most method for hyperbolic decay time series [23]. In the ARFIMA model, the differencing term of ARIMA model is replaced by fractional differencing (d*_f_*), allowing for more flexible fitting to time series with both long-term dependencies and short-term variability [14,15]. For a stationary process, the condition −0.5<d_f_<0.5 holds; if d*_f_* = 0, the series exhibits short-term variability; if −0.5<d*_f_*<0, the series shows intermediate dependence; if 0<d*_f_*<0.5, the series demonstrates long-range dependence properties [7,14]. More specifically, the ARFIMA types can often be represented as ARFIMA (p, d*, q)(P, D*, Q)_S_, where d^∗^ = d + d*_f_*, and −0.5<d *_f_*<0.5 denotes the fractional difference, with d being the integer difference (where d ≥0) [23]. The Hurst exponent (H) provides insights into the persistence of a time series over time [24]. By quantifying the degree of long-term dependence, it helps understanding the underlying dynamics of the series [24]. In application, H is a measure of long-range dependency in a time series, and d*_f_* = H −0.5. The H ranges from 0 to 1, where H = 0.5 suggests an uncorrelated series, 0<H<0.5 implies anti-persistent behaviour, and H>0.5 pinpoints persistent behaviour [14,23]. In this analysis, H was estimated using the rescaled range method (range/standard deviation, R/SD) to determine whether the TB incidence series exhibited long-term dependencies [24]. The ARFIMA model particularly suitable for analysing time series with long-term dependencies [23]. The process of developing an ARFIMA model involves selecting the appropriate modes, as it assumes multiple modes (*i.e*. the fitting function starts the optimisations at multiple starting points), leading to more than one mode [23]. This selection was often determined by maximising the log-likelihood (LL) [23]. The other procedures for estimating parameters and checking the goodness of fit of the ARFIMA model followed the same as the ARIMA model.

### Predictive accuracy measures

Four accuracy measures were used to quantify predictive performance: mean square error (RMSE), mean absolute deviation (MAD), mean error rate (MER), and mean absolute percentage error (MAPE). Lower values of these metrics indicate better predictive performance.

MAD = 
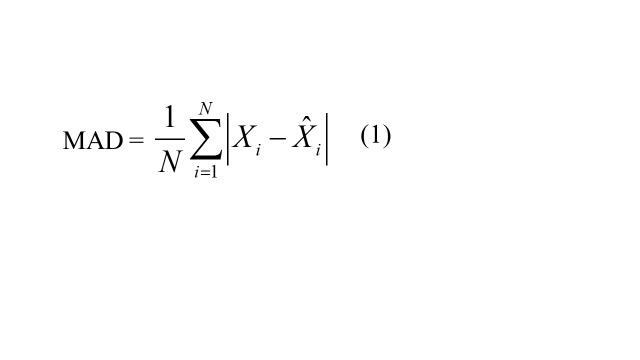
(1)

RMSE = 
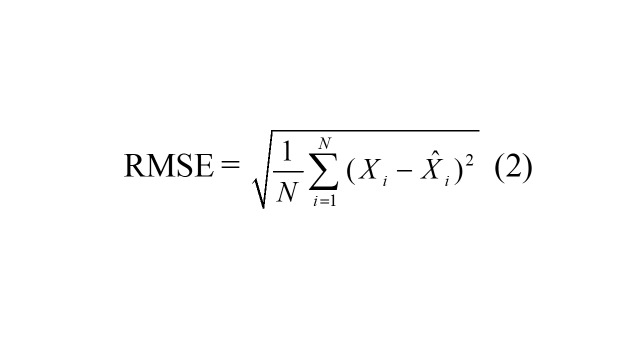
(2)

MAPE = 
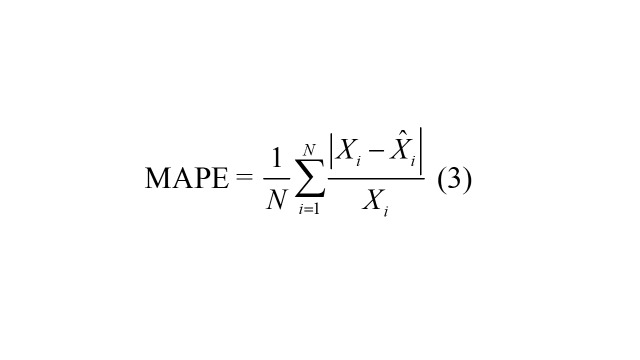
(3)

MER = 
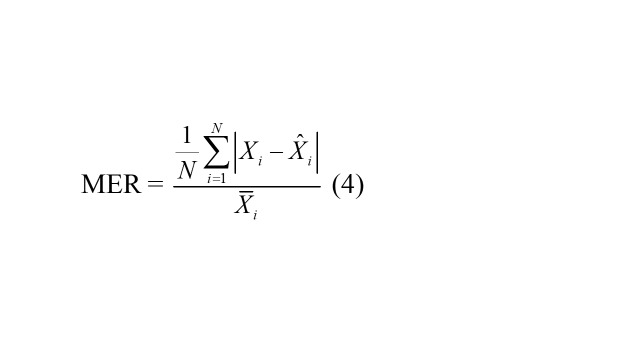
(4)

where, *X_i_* represents the TB observations, signifies the forecasts from the ARIMA and ARFIMA models, denotes the average of TB observations, and *N* is the number of forecasts.

### Statistical analysis

The TB incidence series from January 2007 to May 2022 (185 observations) was treated as the training subset, while the series from June 2022 to May 2023 (12 observations) served as the test subset. The average annual percentage change with a 95% CI was calculated to describe the temporal variation of TB using Joinpoint software (Version 5.0.2, National Cancer Institute, Bethesda, Maryland, USA) [25]. The classical multiplicative decomposition technique was used to investigate the seasonal, trend, and irregular components in the TB incidence series. The ARIMA and ARFIMA models were constructed based on the ‘forecast’ and ‘arfima’ packages, respectively, in *R* (version 3.4.3, *R* Development CoreTeam, Vienna, Austria). The Lagrangian multiplier statistic was used to test the conditional heteroskedastic behaviour and volatility (ARCH effect) in the residuals [26]. Given the established impact of the COVID-19 outbreak on communicable disease epidemics [10,27], a binary variable was incorporated into both models to mitigate the effect of COVID-19 pandemic on the forecasting accuracy, assigning ‘1’ to the period of China's zero-COVID policy (January 2020–December 2022) and ‘0’ to other periods. Additionally, a sensitivity analysis was conducted to evaluate predictive reliability, using the series from January 2007 to December 2022 (192 observations) as the training subsample and reserving the remainder (five observations) as the test subsample. To further compare the predictive performance of the ARIMA and ARFIMA models, we applied the Diebold-Mariano test to assess whether the differences in forecasting errors were statistically significant [28]. Statistical significance was set at a two-sided *P* < 0.05.

## RESULTS

### Data description

**Figure 1:**
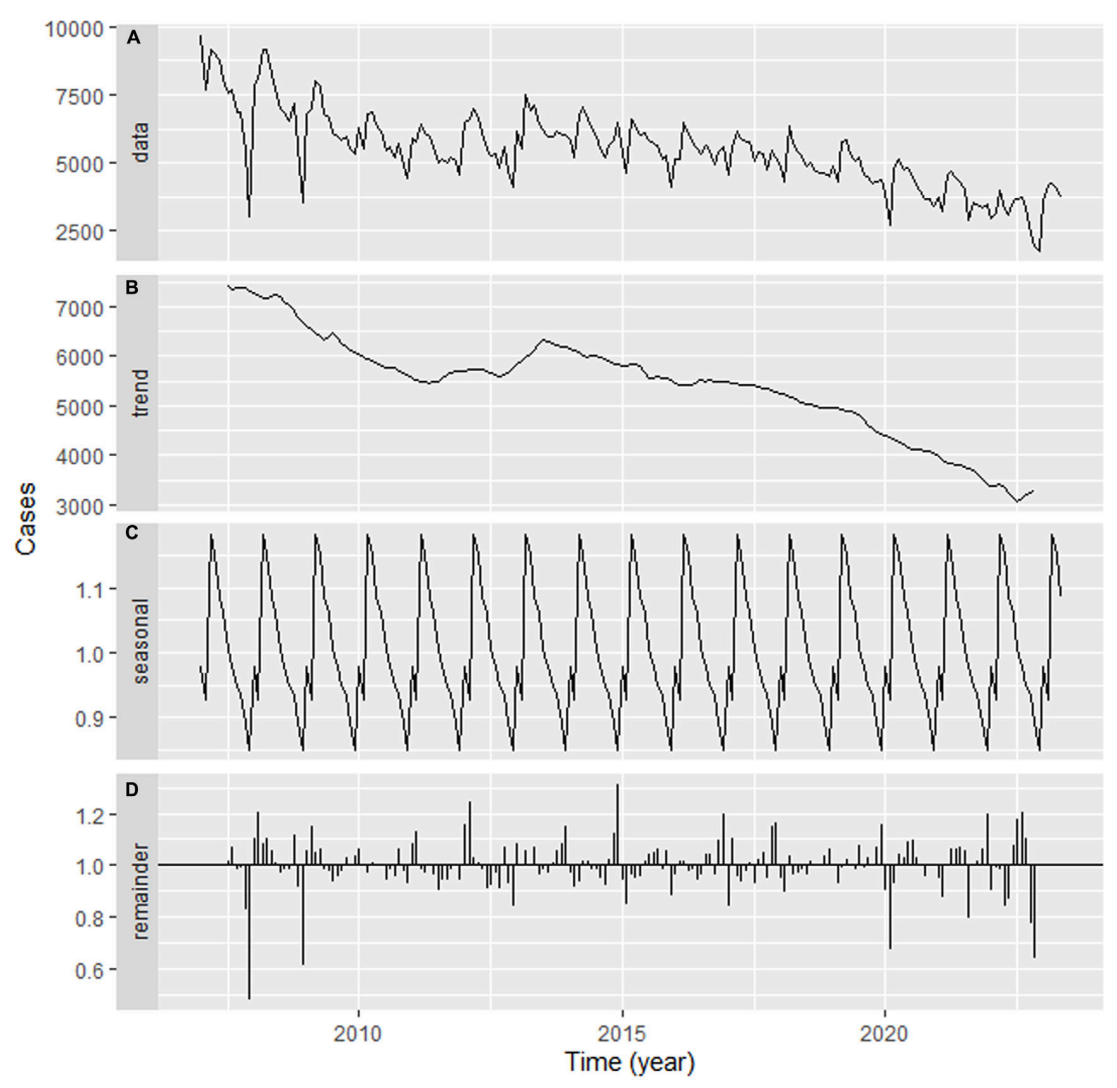
A classical multiplicative decomposition of the TB incidence series in Henan during 2007–2023. **Panel A.** Original series. **Panel B.** Trend. **Panel C.** Seasonality. **Panel D.** Irregular component. The plots display a descending trend and remarkable seasonal behaviour in TB incidence in Henan. TB – tuberculosis.

A total of 1 074 081 TB incident cases were reported in Henan from January 2007 to May 2023, resulting in yearly and monthly morbidity rates of 68.27 and 5.69 per 100 000 persons, respectively. **Figure 1** reports the original series alongside its decomposed components. As depicted in **Figure 1**, Panels A–B, there was a declining trend in TB incidence, with an average annual percentage change of −5.83 (95% CI = −7.98, −3.64; *t* = −5.11, *P* < 0.01). The highest incidence occurred in 2007, with 89 911 cases (95.90 per 100 000 people), which was 1.5 times greater than the lowest recorded level in 2022, when 36 661 cases (37.12 per 100 000 people) were reported. **Figure 1**, Panels C–D illustrate a clear seasonal pattern, with the seasonal factor (SF)>1 between March and July (peaking in March), while SF<1 from August to February (reaching its trough in December) (Figure S1 in the **Online Supplementary Document**).

### The best-fitting ARIMA model

**Figure 2:**
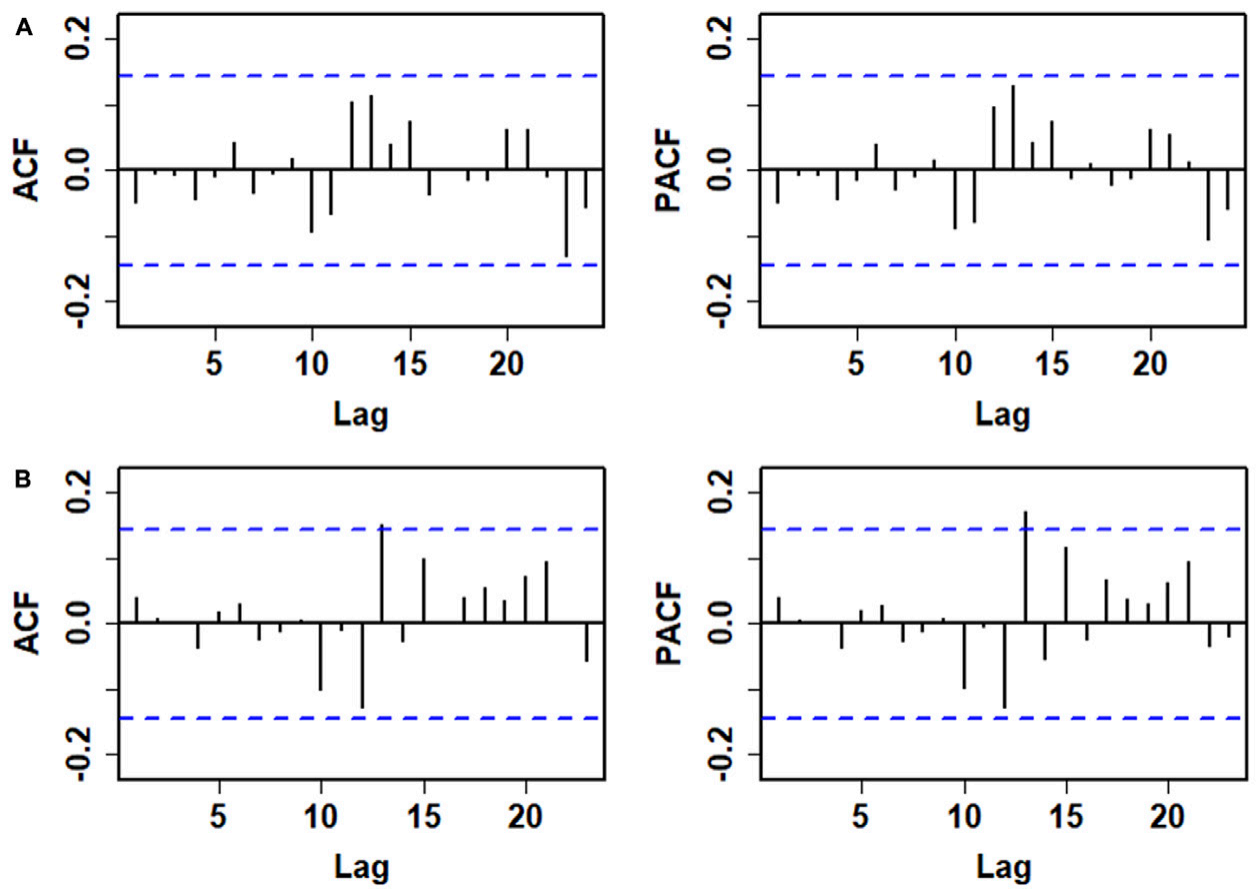
ACF and PACF for the residuals from the models based on the TB incidence series from January 2007 to May 2022. **Panel A.** Auto correlogram and partial auto correlogram from the ARIMA(2,0,1)(1,1,0)_12_ model. **Panel B.** Auto correlogram and partial auto correlogram from the ARFIMA(2,0,1)(1,0.38,0)_12_ model. As depicted in these plots, autocorrelations and partial autocorrelations fall within the threshold limits apart from the one at lag 13 in the ARFIMA model, pinpointing the sufficiency and suitability of both models identified. ACF – autocorrelation function, ARFIMA – autoregressive fractionally integrated moving average, ARIMA – autoregressive integrated moving average, PACF – partial autocorrelation function, TB – tuberculosis.

The ADF test indicated that the TB incidence series was non-stationary (ADF = −1.32, *P* = 0.2). This non-stationarity was addressed through seasonal differencing, resulting in a stationary series (ADF = −5.15, *P* < 0.01). The identification of optimal parameters for the ARIMA model involved examining the ACF and PACF plots of the stationary series. As illustrated in **Table 1**; Figure S2 in the **Online Supplementary Document**, six initial candidate models with significant parameters were identified through trial and error. Subsequent comparisons of information criteria indicated that the ARIMA(2,0,1)(0,1,1)_12_ (An autoregressive model of order 1 (AR1) with a one-month lag  = 1.33 (*t* = 14.30, *P* < 0.01), an autoregressive model of order 2 (AR2) with a two-month lag  = −0.34 (*t* = −3.72, *P* < 0.01), an moving average model of order (MA1) with a one-month lag  = −0.89 (*t* = −18.44, *P* < 0.01), and a seasonal moving average model of order (SMA1) with a one-month lag  = −0.40 ( *t* = −3.99, *P* < 0.01)) specification was preferred, as it minimised AIC (2675.92), corrected AIC (2676.43), and BIC (2694), while maximising LL (−609.26) (**Table 1**). Furthermore, **Figure 2**, Panel A and Table S1 in the **Online Supplementary Document** demonstrate that the residual series exhibited randomness and was uncorrelated with previous values. Besides, the Lagrangian multiplier test revealed the presence of an ARCH effect in the residuals. These assessments confirmed the adequacy of the selected model for fitting the series, allowing it to be employed for forecasting future values from June 2022 to May 2023 (**Table 2**).

**Table 1:** Identified possible ARIMA model with the AIC, CAIC, BIC, and LL values

Models	AIC	CAIC	BIC	LL
ARIMA(2,0,1)(0,1,1)_12_	2675.92	2676.43	2694.84	−1331.96
ARIMA(2,0,1)(1,1,0)_12_	2681.37	2681.87	2700.29	−1334.68
ARIMA(1,0,1)(0,1,1)_12_	2683.29	2683.65	2699.05	−1336.64
ARIMA(1,0,2)(0,1,1)_12_	2676.70	2677.21	2695.62	−1332.62
ARIMA(1,0,2)(1,1,0)_12_	2682.44	2682.95	2701.36	−1335.22
ARIMA(1,0,1)(1,1,0)_12_	2686.48	2686.84	2702.25	−1338.24

AIC – Akaike information criterion, ARIMA – autoregressive integrated moving average, BIC – Bayesian information criterion, CAIC – corrected Akaike information criterion, LL – log-likelihood

**Table 2:** Predictive values from June 2022 to May 2023 under the ARIMA and ARFIMA models

Time	Original observations	ARIMA(2,0,1)(0,1,1)_12_ model		ARFIMA(2,0,1)(1,0.38,0)_12_ model	
		**Forecasts**	**95% CI**	**Forecasts**	**95% CI**
June 2022	3575	3524	2471, 4577	3441	2471, 4942
July 2022	3654	3428	2275, 4581	3359	2275, 4550
August 2022	3698	2691	1507, 3875	2686	1507, 3015
September 2022	3352	3025	1824, 4226	3079	1824, 3648
October 2022	2355	2878	1664, 4092	3019	1664, 3328
November 2022	1903	2819	1594, 4045	2919	1594, 3187
December 2022	1727	2872	1636, 4109	2939	1636, 3272
January 2023	3595	3081	1834, 4328	3583	1834, 3668
February 2023	4164	2904	1647, 4161	3521	1647, 3293
March 2023	4236	4036	2769, 5303	4468	2769, 5537
April 2023	4052	3682	2405, 4959	4002	2405, 4811
May 2023	3736	3442	2156, 4728	3796	2156, 4312

ARFIMA – autoregressive fractionally integrated moving average, ARIMA – autoregressive integrated moving average, CI – confidence interval

### The optimal ARFIMA model

The calculated corrected R/S (Hrs) of 0.96 for the series from January 2007 to May 2022 indicated a significant long-range autocorrelation, thus supporting the application of an ARFIMA model. Then, the parameters p, q, P, and Q were specified in accordance with the ARIMA model, and the fractional differencing order was determined by eliminating models with lower LL value. Table S2 in the **Online Supplementary Document** lists the AIC, BIC, and LL for the candidate modes, indicating that the ARFIMA(2,0,1)(0,0.38,1)_12_ model AR1 = 1.34 (*t* = 14.82, *P* < 0.01), AR2 = −0.34 (*t* = −3.87, *P* < 0.01), MA1 = 0.88 (*t* = 18.51, *P* < 0.01), and SMA1 = −0.27 (*t* = −2.76, *P* < 0.01) specification was preferred due to its maximum LL and minimum AIC and BIC. The residual tests indicated that the residual series behaved like white noise, and the ARCH effects were largely mitigated (**Figure 2**, Panel B, Table S1 in the **Online Supplementary Document**), further validating the appropriateness of the ARFIMA model fitted to the TB incidence series. Consequently, forecasts were generated for the period from June 2022 to May 2023 (**Table 2**).

### Predictive accuracy and reliability

**Figure 3:**
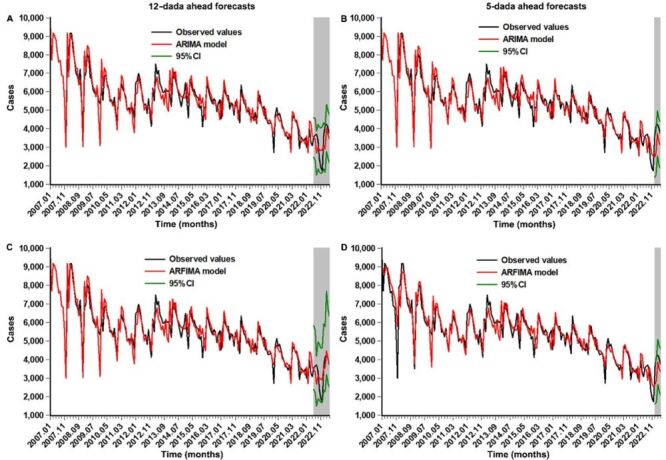
Comparative curves between the observed cases and the forecasts from the ARIMA and ARFIMA models. **Panel A.** 12-data ahead forecasts under the ARIMA(2,0,1)(1,1,0)_12_ model. **Panel B.** 12-data ahead forecasts under the ARFIMA(2,0,1)(1,0.38,0)_12_ model. **Panel C.** 5-data ahead forecasts under the ARIMA(2,0,1)(1,1,0)_12_ model. **Panel D.** 5-data ahead forecasts under the ARFIMA(2,0,1)(1,-0.34,0)_12_ model. In plots, the grey shaded area represents the predictive values with their 95% CI. We note that the forecasts from the ARFIMA model are seemingly in closer proximity to the actual curve than those from the ARIMA model in both 12- and 5-step ahead forecasts. ARFIMA – autoregressive fractionally integrated moving average, ARIMA – autoregressive integrated moving average, CI – confidence interval.

Following the construction of the ARIMA and ARFIMA models, the ARIMA(2,0,1)(1,1,0)_12_ and ARFIMA(2,0,1)(1, −0.37,0)_12_ specifications were selected as the best-fitting models in the sensitivity analysis (Hrs = 0.96). Table S3–5 and Figure S3 in the **Online Supplementary Document** summarise the selected parameters, goodness of fit, and forecasts for both models. **Table 3** and **Figure 3** compare the predictive accuracy and reliability across different data sets. Notably, the ARFIMA model generated smaller predictive errors compared to the ARIMA model, with the resulting Diebold-Mariano statistics indicating statistical significance (*P* < 0.05). Sensitivity analysis further corroborated the robustness of these findings. These results indicate that the ARFIMA model outperforms the ARIMA model in forecasting TB incidence in Henan, effectively capturing long-term dependencies and seasonal patterns.

**Table 3:** Comparison of the fitting and predictive accuracy measurement metrics under the ARIMA and ARFIMA models

Metrics	Fitting part		Predictive part		DM statistic	*P-*value
	**ARIMA model**	**ARFIMA model**	**ARIMA model**	**ARFIMA model**		
12-data ahead forecasts						
MAD	385.07	368.96	569.54	467.00	3.42	<0.01
MAPE	0.07	0.07	0.21	0.19	2.74	0.01
RMSE	524.87	524.22	690.11	620.48	2.40	0.02
MER	0.07	0.07	0.17	0.14	—	—
5-data ahead forecasts						
MAD	391.70	372.82	899.59	705.58	27.52	<0.01
MAPE	0.08	0.08	0.23	0.18	27.00	<0.01
RMSE	544.02	528.12	970.95	789.59	11.72	<0.01
MER	0.07	0.07	0.23	0.18	—	—

ARFIMA – autoregressive fractionally integrated moving average, ARIMA – autoregressive integrated moving average, DM – Diebold-Mariano, MAD –mean absolute deviation, MAPE – mean absolute percentage error, MER – mean error rate, RMSE – mean square error

## DISCUSSION

This study is the first to explore the flexibility and sufficiency of the ARFIMA model in forecasting TB epidemics, particularly by considering long-term dependencies. Our findings indicated that the ARFIMA model was adequate for forecasting TB epidemics, as it more accurately and reliably captures various patterns and dynamics in forecasting compared to the ARIMA model. This matches well with earlier literature that has demonstrated the satisfactory predictive capabilities of the ARFIMA model in fields such as finance, economics, and meteorology [14,16,18,23]. Also, our results share similarity with two recent studies in the field of health, which have shown that the ARFIMA model enhances forecasting accuracy for conditions such as haemorrhagic fever with renal syndrome and emergent infection [7,29], further reinforcing its flexibility and usefulness in public health contexts. Furthermore, the ARFIMA model can also be deemed as a tool for monitoring and evaluating the effectiveness of implemented interventions. By comparing the forecasts with the actual series, policymakers can assess the suitability of the strategies and make necessary adjustments if required. This iterative process ensures a dynamic and adaptive approach to epidemic prevention and control.

The analysis revealed a declining trend in TB incidence in Henan, with an annual reduction rate of approximately 5.83%. This trend is consistent with the broader patterns observed in China and globally [1,30]; however, it is notably higher than the national average reduction of about 3% per year since 2005 [30]. This suggests that the comprehensive implementation of the directly observed treatment of short-course strategy and other TB control measures have had a positive impact in Henan. Nonetheless, recent studies indicate that the decline in TB incidence is facing challenges due to evolving epidemiological patterns and risk factors, including the aging population and the emergence of drug-resistant TB [1–3]. These factors complicate efforts to meet the ambitious targets set for TB elimination, which call for a 10% annual decline from 2020 to 2025 and a 17% decline from 2025 to 2035 [31].

Seasonality is a critical characteristic of infectious disease epidemics, and our study identified a clear seasonal pattern in Henan. This finding corroborates previous research indicating that TB exhibits seasonal fluctuations, with peaks occurring in late winter and spring and troughs in autumn and early winter [32]. Such seasonal dynamics are consistent with findings from most studies in China and USA, South Korea, Mongolia, Japan, and Kuwait [32–34], although they differ from observations in Spain (peak in June) [35], Japan (dual peaks in June and October) [36], and Iraq (peak in spring and winter) [37]. This is mainly related to the geographical location, climate change, and socio-economic conditions of each country. The seasonal peak and trough in TB incidence are mainly e attributable to the ‘Spring Festival effect’ in China.

The ARIMA model has been widely used for analysing morbidity and mortality trends of infectious diseases (*e.g.* TB, haemorrhagic fever with renal syndrome, *Brucellosis*, Scarlet fever, COVID-19) owing to its flexibility in accommodating changing trends, periodic variations, and random fluctuations, ensuring relatively accurate forecasts [5,8,10–13]. Another advantage of ARIMA model is the relatively straightforward explanation to end-users, as it fails to rely heavily on complex mathematical concepts. However, the ARIMA model assumes that the forecasts follow a linear AR process and it only captures short-term randomness in a series and may result in an over-difference for series [7], which may limit the effective application in TB incidence series with clear long-range dependency. This also matches well with prior studies that the ARIMA model is effective for short-term forecasts [5]. In contrast, the ARFIMA model offers a robust framework for modelling time series that effectively integrates the advantages of ARIMA and while allowing for fractional differencing, making it particularly suitable for capturing long-range dependencies in data [14,15]. This methodology presents several advantages over traditional ARIMA models. First, ARFIMA model accommodates both short- and long-run dependencies within a time series, which enhances the reliability and accuracy of forecasts [23]. Second, its capacity to handle outliers and extreme values through the incorporation of long-range dependence properties contributes to a more resilient and robust modelling approach [23]. Finally, fractional differencing equips ARFIMA model with a greater ability to capture seasonal patterns in a series compared to ARIMA model [7]. These advantages elucidate why ARFIMA model yields more accurate and reliable forecasts in TB incidence series than ARIMA model. Notwithstanding the usefulness and flexibility of the ARFIMA model, it should be noted that if our findings require to be transferable to other infectious diseases, further work will need to be done as the data-generating mechanism exhibits a high level of complexity and diversity. Besides, recent work has indicated successful applications of some advanced methods, including nonlinear autoregressive neural network [38], Bayesian structural time series technique [10], Elman and Jordan neural network [6]. Therefore, to optimise the forecasting accuracy for TB, further studies involve comparing the forecasting ability of ARFIMA model with the ones above.

Despite the advantages of the ARFIMA model, several limitations must be acknowledged. First, the TB incident cases were derived from a passive monitoring system, which may result in underdiagnosis and underreporting. Second, while our findings demonstrate the ARFIMA model’s efficacy in forecasting TB epidemics, further research is needed to assess its applicability to other infectious diseases, given the complexity and diversity of data-generating mechanisms. Third, to ensure the accuracy of forecasts, it is essential to update the TB incidence series regularly with new case data. Fourth, the quality of parameter estimates in the ARFIMA model is influenced by the length of the data series [7]; previous studies suggest that a minimum of 100 observations is recommended for effective modelling [39]. Finally, the current study was limited to monthly TB case counts from the Health Commission of Henan, which precluded detailed demographic analyses based on variables such as age, gender, and urban-rural distribution. Future research could benefit from integrating TB-associated risk factors into the ARFIMA model to enhance forecasting accuracy.

## CONCLUSIONS

In sum, our study indicates that TB incidence in Henan exhibits a clear downward trend alongside notable seasonal pattern. The ARFIMA model demonstrates superior accuracy and reliability in forecasting TB incidence compared to the ARIMA model. Employing the ARFIMA model for forecasting TB trends at the population level in Henan represents a valuable approach for understanding disease dynamics, guiding public health planning, and monitoring progress towards TB control objectives. Its effectiveness in capturing seasonal and long-term trends is critical for resource allocation and early detection of changes in disease dynamics.

## Additional material


Online Supplementary Document
