# Supplementary material for: Forecasting tuberculosis epidemics using an autoregressive fractionally integrated moving average model: a 17-year time series analysis
Source: J Glob Health. 2025 Jul 25;15:04208. doi: 10.7189/jogh.15.04208 (PMC12580775; doi:10.7189/jogh.15.04208)
Supplement: Online Supplementary Document [file jogh-15-04208-s001.pdf]

Supplement to: Wang Y, Liang Y, Zhang B, Yi S, Zhou P, Lan X, Xue C, Li Y, Li X, Xu C. Forecasting tuberculosis epidemics using an autoregressive fractionally integrated moving average model: a 17-year time series analysis. J Glob Health. 2025;15:04208.

Table S1 Box-Ljung *Q* statistics for the residuals from the ARIMA and ARFIMA models

**Lags**	**ARIMA(2,0,1)(0,1,1)$_{12}$ model**				**ARFIMA(2,0,1)(1,0.38,0)$_{12}$ model**			
	**Q**	***P***	**LM**	***P***	**Q**	***P***	**LM**	***P***
1	0.45	0.50	22.24	< 0.01	0.32	0.57	9.05	< 0.01
3	0.46	0.93	23.14	< 0.01	0.33	0.95	10.53	0.02
6	1.18	0.98	24.26	< 0.01	0.81	0.99	11.24	0.08
9	1.45	1.00	24.48	< 0.01	0.95	1.00	11.29	0.26
12	6.17	0.91	36.37	< 0.01	6.19	0.91	14.29	0.28
15	10.21	0.81	39.24	0.01	13.03	0.60	24.43	0.06
18	10.52	0.91	39.37	< 0.01	13.99	0.73	25.38	0.12
21	12.19	0.93	39.45	< 0.01	17.23	0.70	25.89	0.21
24	16.53	0.86	44.18	< 0.01	19.74	0.71	48.79	< 0.01
27	23.44	0.66	47.75	< 0.01	26.75	0.48	49.40	< 0.01
30	26.55	0.65	48.65	0.02	28.57	0.54	49.91	0.01
33	28.46	0.69	48.52	0.04	29.78	0.63	51.87	0.02
36	29.37	0.78	60.57	< 0.01	30.21	0.74	45.76	0.13

ARFIMA – autoregressive fractionally integrated moving average, ARIMA – autoregressive integrated moving average, LM – Lagrangian multiplier statistic, Q – Ljung-Box Q statistic

Table S2 The resulting modes with the AIC, BIC, and LL values

Modes	AIC	BIC	LL
Mode 1	2353.82	2382.80	-1167.91
Mode 2	2354.05	2383.04	-1168.03
Mode 3	2354.10	2383.08	-1168.05
Mode 4	2355.83	2384.81	-1168.92
Mode 5	2355.89	2384.88	-1168.95
Mode 6	2357.10	2386.08	-1169.55
Mode 7	2357.62	2386.60	-1169.81
Mode 8	2361.59	2390.57	-1171.79
Mode 9	2363.72	2392.71	-1172.86
Mode 10	2366.69	2395.67	-1174.35
Mode 11	2366.69	2395.68	-1174.35
Mode 12	2366.70	2395.68	-1174.35
Mode 13	2366.70	2395.68	-1174.35
Mode 14	2366.70	2395.68	-1174.35
Mode 15	2366.70	2395.68	-1174.35
Mode 16	2376.64	2405.63	-1179.32

AIC, Akaike information criterion; BIC, Bayesian information criterion, LL, log-likelihood.

Table S3 The identified parameters of the best ARIMA and ARFIMA model in the sensitivity analysis

Variables	Estimates	Standard error	t	*P*
ARIMA(2,0,1)(1,1,0)$_{12}$ method developed on data from January 2007 to December 2022				
AR1	1.35	0.09	14.79	<0.01
AR2	-0.36	0.09	-4.10	<0.01
MA1	-0.90	0.05	-18.86	<0.01
SAR1	-0.26	0.08	-3.20	0.01
ARFIMA(2,0,1)(1,-0.37,0)$_{12}$ method developed on data from January 2007 to December 2022				
AR1	1.34	0.09	14.82	<0.01
AR2	-0.34	0.09	-3.87	0.01
MA1	0.88	0.05	18.51	<0.01
SAR1	-0.27	0.10	-2.76	0.01

ARIMA, autoregressive integrated moving average; AR1, moving average at a 1-month lag; AR2, moving average at a 2-month lag; MA1, moving average at a 1-month lag; SAR1, seasonal moving average at a 12-month lag.

Table S4 Predictive values from January 2023 to May 2023 under the ARIMA and ARFIMA

Time	Original observations	ARIMA		ARFIMA	
		Forecasts	95%*CI*	Forecasts	95%*CI*
2023-January	3595	2467	1385 - 3548	2680	1622 - 3737
2023-February	4164	2669	1482 - 3856	2866	1693 - 4039
2023-March	4236	3743	2525 - 4961	3875	2666 - 5085
2023-April	4052	3300	2067 - 4533	3506	2278 - 4735
2023-May	3736	3106	1863 - 4350	3328	2086 - 4570

ARIMA, autoregressive integrated moving average; ARFIMA, autoregressive fractionally integrated moving average; *CI*, confidence interval.

Table S5 The resulting modes with the AIC, BIC, and LL values

Modes	AIC	BIC	LL
Mode 1	2442.09	2471.41	-1212.05
Mode 2	2443.12	2472.43	-1212.56
Mode 3	2444.94	2474.25	-1213.47
Mode 4	2445.48	2474.79	-1213.74
Mode 5	2445.87	2475.19	-1213.94
Mode 6	2446.87	2476.18	-1214.43
Mode 7	2446.87	2476.19	-1214.43
Mode 8	2446.91	2476.23	-1214.46
Mode 9	2447.94	2477.25	-1214.97
Mode 10	2448.96	2478.28	-1215.48
Mode 11	2449.59	2478.90	-1215.79
Mode 12	2449.97	2479.28	-1215.98
Mode 13	2450.23	2479.55	-1216.11
Mode 14	2450.34	2479.66	-1216.17
Mode 15	2450.53	2479.84	-1216.26
Mode 16	2450.77	2480.09	-1216.39
Mode 17	2452.60	2481.92	-1217.30
Mode 18	2452.97	2482.28	-1217.48
Mode 19	2453.91	2483.22	-1217.95
Mode 20	2453.92	2483.24	-1217.96
Mode 21	2454.18	2483.50	-1218.09
Mode 22	2455.70	2485.01	-1218.85
Mode 23	2456.46	2485.78	-1219.23
Mode 24	2457.56	2486.88	-1219.78
Mode 25	2460.63	2489.95	-1221.31
Mode 26	2461.25	2490.57	-1221.62
Mode 27	2461.28	2490.59	-1221.64
Mode 28	2461.28	2490.60	-1221.64
Mode 29	2469.29	2498.61	-1225.64

AIC, Akaike information criterion; BIC, Bayesian information criterion, LL, log-likelihood.

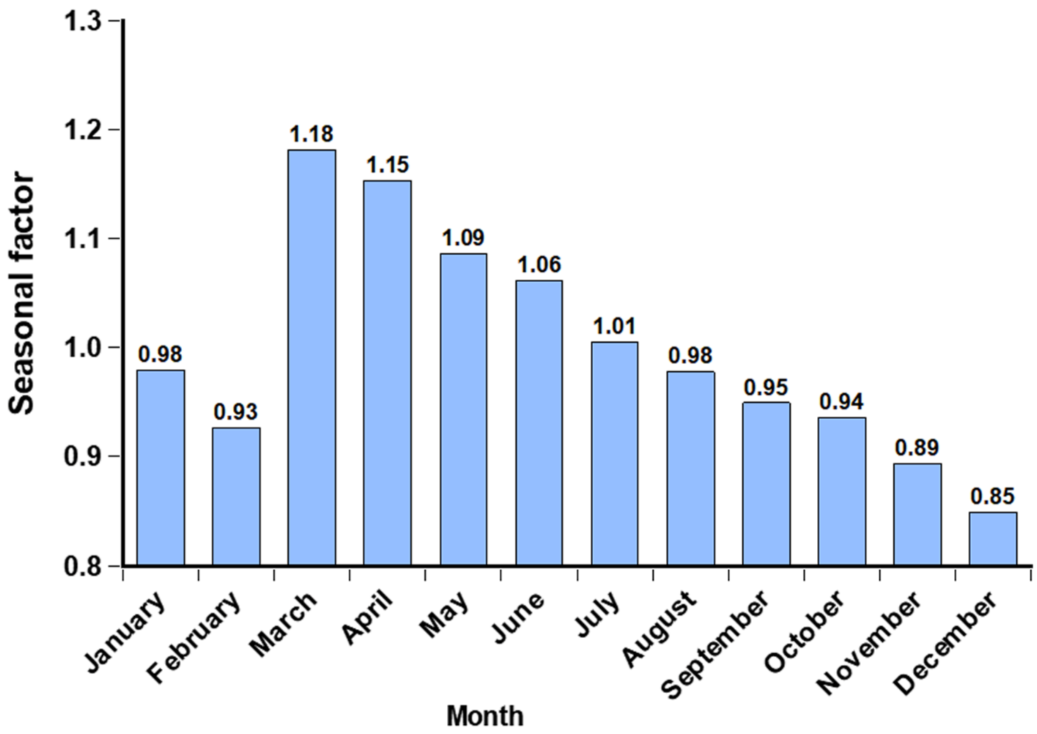

Figure S1. Seasonal behaviour of the TB incidence series in Henan during 2007–2023. Often, the seasonal factor is > 1, indicating a high-risk seasonality; otherwise not. It can be seen from this plot that a notable seasonality with a peak in March and a trough in December. TB – tuberculosis.

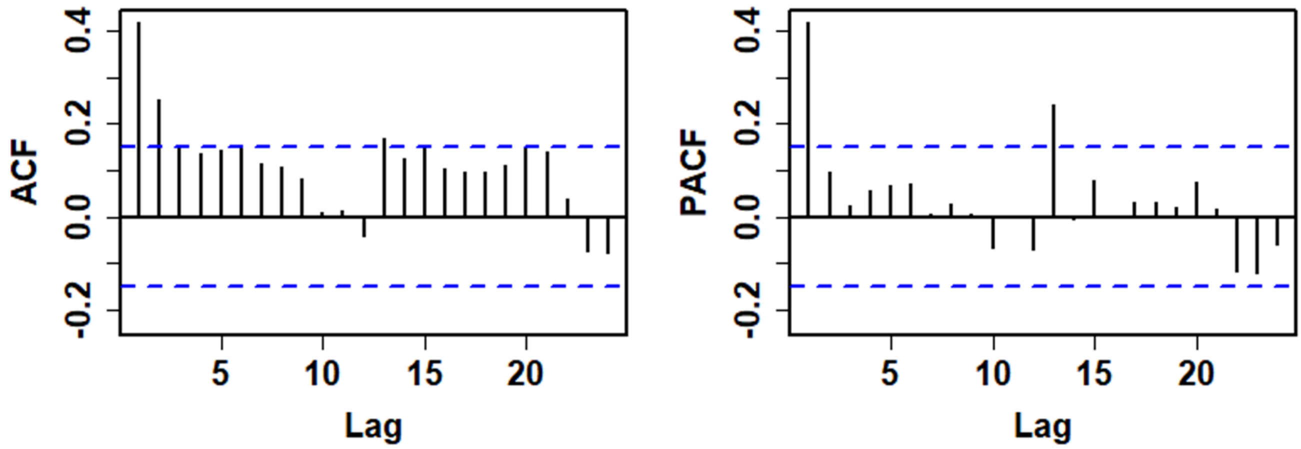

Figure S2. Autocorrelation function (ACF) and partial autocorrelation function (PACF) plots for the seasonally differenced TB incidence series from January 2007 to May 2022. The ACF and PACF shown in this plot may be suggestive of the most orders of the SMA being two, and the most orders of the SAR being one. Thereafter, some possible models were tried based on the crudely selected orders selected until the best possible model with the lowest AIC, CAIC, or BIC, or with the largest LL was discovered.

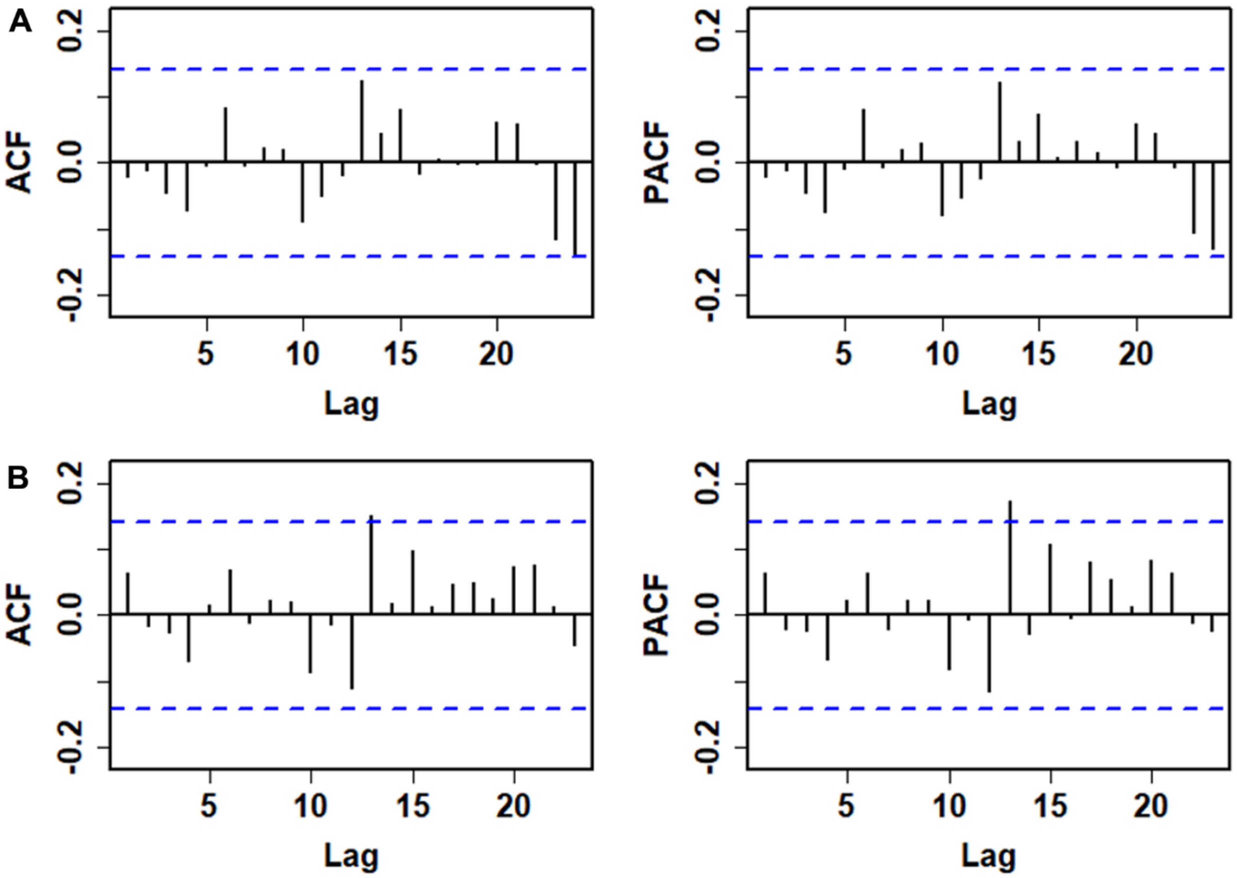

Figure S3. Autocorrelation function (ACF) and partial autocorrelation function (PACF) plots for the residuals from the model based on the TB incidence series from January 2007 to December 2022. (A) autocorrelogram and partial autocorrelogram from the ARIMA(2,0,1)(1,1,0)$_{12}$, (B) autocorrelogram and partial autocorrelogram from the ARFIMA(2,0,1)(1,-0.37,0)$_{12}$. As depicted in these plots, autocorrelations and partial autocorrelations fall within the threshold limits apart from the one at lag 13 in the ARFIMA, pinpointing the sufficiency and suitability of both models identified.
